# Nanomaterial Composite Compatibilized Rubber–Plastic Elastomer–Asphalt Interface Mechanism and Performance Evaluation

**DOI:** 10.3390/ma19091857

**Published:** 2026-04-30

**Authors:** Tangxin Xie, Zhongming He, Jue Li, Chao Huang, Pengxu Wang, Qiao Zhao

**Affiliations:** 1 School of Traffic and Transportation on Engineering, Changsha University of Science & Technology, Changsha 410004, China; xtx96@csust.edu.cn (T.X.); hezhongming45@126.com (Z.H.); 23201060266@csust.edu.cn (P.W.); 23201060294@csust.edu.cn (Q.Z.); 2 National Engineering Research Center of Highway Maintenance Technology, Changsha 410004, China; 3 School of Civil Engineering, Central South University, Changsha 410075, China; huangchao-22@csu.edu.cn

**Keywords:** nanomaterials, rubber–plastic-based elastomer, modified asphalt, interface compatibility, high-temperature rheology, fatigue performance, low-temperature crack resistance

## Abstract

Conventional rubber–plastic modified asphalt often suffers from poor compatibility and thermal storage stability, which limits its engineering application. To address this issue, this study proposes a prefabricated nano-reinforced rubber–plastic thermoplastic elastomer (TPE) modification strategy. The specific objective was to comparatively investigate how different waste plastic matrices (HDPE, LDPE, and PP) and two representative nano-oxides (ZnO and TiO_2_) affect the interfacial evolution, storage stability, rutting resistance, fatigue durability, and low-temperature cracking resistance of modified asphalt. The prefabricated nano-reinforced TPE modifier was incorporated into the base asphalt, and its storage stability, interface evolution and multi-scale rheological properties were evaluated. The results show that all modified binders exhibited good thermal storage stability, with softening point differences below 2.5 °C. The enhancement mechanism was mainly governed by physical blending, swelling adsorption, and interfacial synergistic interactions rather than the formation of new chemical functional groups. A clear synergistic matching relationship between plastic type and nanoparticle type was identified. LDPE-based systems showed better phase compatibility and fatigue/low-temperature performance, whereas HDPE-based systems were more favorable with respect to improvement of high-temperature rutting resistance. In addition, ZnO contributed more significantly to storage stability, rutting resistance, and fatigue resistance, while TiO_2_ was more beneficial for low-temperature crack resistance. These findings provide new insight into the interfacial design of nano-reinforced rubber–plastic modified asphalt and offer guidance for performance-oriented and sustainable pavement materials.

## 1. Introduction

With the rapid growth of highway traffic and the increasing frequency of extreme climate events, conventional-base asphalt can no longer meet the performance demands of modern pavement [1]. Under heavy traffic and complex service conditions, asphalt binders must provide sufficient resistance to rutting at high temperature, cracking at low temperature, and fatigue under repeated loading [2]. For this reason, polymer-modified asphalt has been widely used in pavement engineering. Previous studies have demonstrated that the combined use of waste rubber and waste plastics can significantly improve the high-temperature deformation resistance, mechanical performance, and aging resistance of asphalt [3,4]. These findings confirm the feasibility of a rubber–plastic composite modification and highlight its potential for sustainable pavement construction [5,6]. However, they also indicate that the overall effectiveness of such modification depends not only on the incorporation of multiple polymers, but also on whether the multiphase system can maintain sufficient compatibility, uniform dispersion, and thermal storage stability [7]. Zhu et al. [8] pointed out that good modification performance relies on the fine and homogeneous dispersion of polymers in the continuous asphalt phase, while Wang et al. [9] emphasized that poor storage stability at high temperature remains one of the major factors limiting the large-scale application of composite polymer-modified asphalt. These studies are closely related to the present work, because both previous studies and the current investigation focus on the compatibility problem in rubber–plastic-modified asphalt. Nevertheless, most previous studies mainly examined conventional direct blending systems or general rubber–plastic alloy modified asphalt, whereas the present study adopts a prefabricated rubber–plastic TPE route to improve phase regulation and interfacial stability.

Nanomaterials have been widely recognized as a promising means of enhancing asphalt performance because of their high specific surface area, high surface energy, and strong interfacial activity [10,11]. Previous studies showed that nano ZnO and nano TiO_2_ can improve the thermal stability, rheological behavior, and aging resistance of asphalt, while nano inorganic particles can also strengthen the interface between modifiers and asphalt, thereby reducing phase separation and improving storage stability [12,13]. Owing to their established technical maturity and successful commercialization, these two oxides have become the most prevalent nanomaterials in asphalt modification. Their increasingly competitive cost due to mass production further justifies their selection for enhancing the rubber–plastic asphalt system. These findings suggest that nanomaterials may serve not only as performance enhancers, but also as effective interfacial regulators in multiphase asphalt systems. This is similar to the objective of the present study, which also aims to improve the compatibility and performance of rubber–plastic-modified asphalt through interfacial regulation [14]. However, different from previous studies that mainly focused on conventional polymer-modified asphalt or discussed the general effects of nanomaterials, the present investigation further examines how different plastic matrices (HDPE, LDPE, and PP) interact with different nanoparticle types (ZnO and TiO_2_) in a prefabricated nano-reinforced rubber–plastic TPE system. Therefore, this study is intended to clarify the similarities and differences in interfacial evolution and rheological performance among these systems, and to reveal the synergistic matching relationship between plastic type and nanoparticle type.

The aim of this study is to investigate the effects of different plastic matrices and nanomaterials on the interfacial mechanism and engineering performance of composite modified asphalt prepared by this route. Softening point difference, fluorescence microscopy (FM), and Fourier transform infrared spectroscopy (FTIR) were used to characterize storage stability, phase distribution, interfacial evolution, and physicochemical interaction. In addition, multiple stress creep recovery (MSCR), linear amplitude sweep (LAS), and bending beam rheometer (BBR) tests were carried out to evaluate high-temperature rutting resistance, fatigue durability, and low-temperature cracking resistance. This study is expected to provide a clearer understanding of the interfacial synergistic mechanism in nano-reinforced rubber–plastic composite asphalt. It may also provide useful support for the design and application of high-performance and sustainable modified asphalt.

## 2. Test Raw Materials

### 2.1. Materials

The matrix asphalt used in this study was Sinopec 70# road petroleum asphalt. Its basic properties are listed in Table 1. Low Mooney reclaimed rubber was used as the rubber phase. It was produced from desulfurized rubber powder through physical treatment, mechanical shearing, and chemical reactions. These processes further destroyed the internal crosslinked network of rubber and then the material was re-plasticized and reformed [15]. Its main properties are shown in Table 2. Three waste plastics, including high-density polyethylene (HDPE), low-density polyethylene (LDPE), and polypropylene (PP), were used as the plastic phase of the elastomer. Their main physicochemical properties are presented in Table 3. Nano ZnO and nano TiO_2_, which are commonly used in asphalt modification, were selected as the nanomaterials. Their main physicochemical properties are listed in Table 4. Maleic anhydride grafted compatibilizer FG1901 was used as the compatibilizer, and its main physicochemical properties are given in Table 5. The basic physicochemical properties of the selected nanoparticles, including average particle size, purity, specific surface area, and bulk density, are summarized in Table 4. Their dispersion state and interfacial influence on the modified binders were subsequently evaluated through fluorescence microscopy (FM), Fourier transform infrared spectroscopy (FTIR), and the corresponding rheological responses.

### 2.2. Preparation of Nano-Reinforced Rubber–Plastic Elastomer

**Figure 1:**
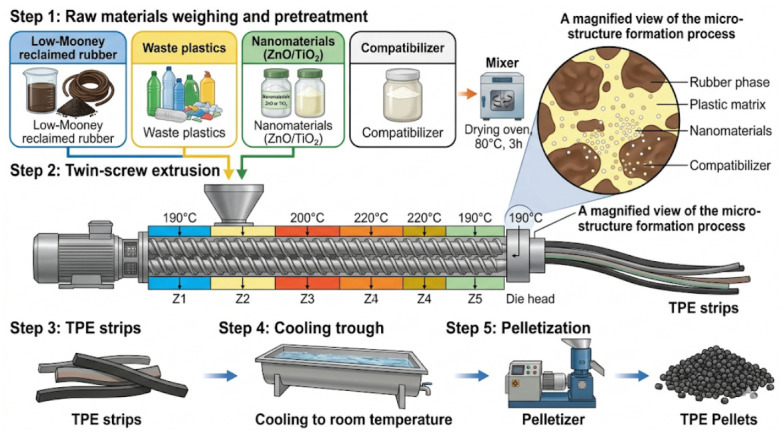
Preparation process of nano-compatibilized rubber–plastic elastomer.

To improve the compatibility of nano rubber–plastic-modified asphalt, TPE particles were prepared using a twin-screw extrusion process. Low Mooney reclaimed rubber and waste plastics were melt blended in the presence of a compatibilizer to form a continuous polymer matrix, while nano ZnO or nano TiO_2_ were introduced into the polymer phase. Considering the strong tendency of nanoparticles to agglomerate because of their high specific surface area and surface energy, the nanoparticles were incorporated into the rubber–plastic matrix during melt blending rather than being directly added into asphalt. The combined effects of pre-mixing, compatibilizer-assisted interfacial regulation, and continuous thermal–shear dispersion in the twin-screw extruder were expected to improve the dispersion of nanoparticles in the polymer phase and to reduce severe agglomeration. In addition, the subsequent high-speed shearing during asphalt preparation further promoted the redistribution of the prefabricated nano-reinforced TPE particles in the asphalt matrix. Considering the strong tendency of nanoparticles to agglomerate because of their high specific surface area and surface energy, the nanoparticles were incorporated into the rubber–plastic matrix during melt blending rather than being directly added into asphalt. The combined effects of pre-mixing, compatibilizer-assisted interfacial regulation, and continuous thermal–shear dispersion in the twin-screw extruder were expected to improve the dispersion of nanoparticles in the polymer phase and to reduce severe agglomeration. In addition, the subsequent high-speed shearing during asphalt preparation further promoted the redistribution of the prefabricated nano-reinforced TPE particles in the asphalt matrix. Low Mooney reclaimed rubber and waste plastics were melt blended in the presence of a compatibilizer to form a continuous polymer matrix, while nano ZnO or nano TiO_2_ were dispersed into the polymer phase. The detailed preparation procedure is shown in Figure 1.

(1) Low Mooney reclaimed rubber, waste plastics, nanomaterials, and compatibilizer were weighed according to Table 5. All raw materials were dried at 80 °C for 3 h and then mixed uniformly.
(2) The mixed materials were fed into a twin-screw extruder. The temperatures of the five zones were set to 190, 200, 220, 220, and 190 °C, respectively, and the die head temperature was also set to 190 °C. The main screw speed was 12 r/min, and the feeder speed was 15 r/min. The extruded TPE strips were cooled to room temperature and solidified.
(3) The cooled TPE strips were then pelletized into particles of suitable size.

### 2.3. Modified Asphalt Preparation Method

**Figure 2:**
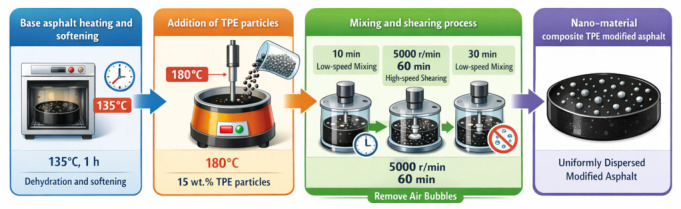
Preparation process of modified asphalt.

A modification route based on the pre-fabrication of TPE particles and their subsequent external incorporation into asphalt was used in this study. The nanomaterials were first dispersed and fixed in the TPE particles, and then the prepared particles were added into the base asphalt to produce the final modified binders. The specific preparation flow chart is shown in Figure 2, and the specific matching of each modified asphalt is shown in Table 5.

The composition of the nano rubber–plastic TPE modifiers was kept constant among the different groups to ensure comparability of the study, with only the plastic type (HDPE, LDPE, or PP) and nanoparticle type (ZnO or TiO_2_) being varied. The selected rubber/plastic ratio, compatibilizer dosage, and nanoparticle dosage were determined based on preliminary formulation design, relevant previous studies, and processing feasibility during extrusion. In this work, the concentrations were not intended to represent a universal optimum for all systems; instead, they were chosen as fixed and representative levels to enable a controlled comparison of the effects of plastic matrix type and nanoparticle type on interfacial evolution and binder performance.

(1) The base asphalt was heated in an oven at 135 °C for about 1 h until it became soft and fluid. This step was used for dehydration and softening.
(2) The heated asphalt was transferred into a thermostatic heating mantle and kept at 180 °C. A high-speed shear mixer was inserted into the asphalt, and 15 wt.% TPE particles were then added. A fixed TPE dosage of 15 wt.% was adopted for all binders to maintain a consistent comparison basis among different formulations while ensuring that the modifier content was high enough to produce measurable changes in storage stability, interfacial characteristics, and rheological performance.
(3) The binder was first mixed at low speed for 10 min to promote the initial dispersion and swelling of the modifier. Then, high-speed shearing was carried out at 5000 r/min for 60 min. After that, low-speed mixing was continued for 30 min to remove air bubbles.

Note: The TPE material ratio (70:30:5:30) represents the internal mass parts of rubber, plastic, compatibilizer, and nanomaterials. The final modified asphalt was prepared by adding 15 wt.% of these pellets to the base asphalt. For all modified binders, the dosage of the prefabricated TPE modifier was fixed at 15 wt.% of the base asphalt in order to ensure comparability among different formulations. Therefore, the present study focuses on comparative evaluation under a constant dosage condition, rather than on dosage optimization.

## 3. Test Scheme

### 3.1. Storage Stability Test

Storage stability was evaluated according to JTG E20-2011 [16]. The modified binders were stored in segregation tubes at 163 ± 5 °C for 48 h and then cut into upper and lower sections after cooling. The softening point difference between the two sections (ΔSP) was used to assess thermal storage stability. A smaller ΔSP value indicates a lower segregation tendency and better storage stability of the modified binder during high-temperature storage.

### 3.2. Fluorescence Microscopy (FM)

FM was used to observe the dispersion morphology of the polymer-rich phase in the modified binders. A small amount of hot binder was spread on a glass slide to form a thin film and then cooled to room temperature. The images were recorded using a fluorescence microscope (Fluorescence microscope Axio Imager M2, Zeiss, Oberkochen, Germany) at ×100 magnification. The size, distribution, and continuity of the fluorescent phase were used to qualitatively evaluate the compatibility and dispersion uniformity of the modifier in the asphalt matrix.

### 3.3. Fourier Transform Infrared Spectroscopy (FTIR)

FTIR was performed on the base and modified binders using an FTIR spectrometer (Infrared spectrometer ALPHA II, Bruker, Ettlingen, Germany) in ATR mode. Spectra were collected over 4000–400 cm^−1^ at a resolution of 4 cm^−1^ with 32 scans for each sample. The obtained spectra were used to compare the characteristic functional groups of different binders and to analyze whether the modification mainly involved chemical interaction or physical blending.

### 3.4. Dynamic Shear Rheometer Tests

Rheological tests were conducted using a DSR (DSR SmartPave302, Anton Paar, Graz, Austria). The temperature sweep test was performed according to AASHTO T315 [17] with a 25 mm plate, 1 mm gap, 10 rad/s frequency, and 1% strain. The MSCR test was carried out according to AASHTO T350-19 [18] at 64 °C under 0.1 and 3.2 kPa, with 1 s creep and 9 s recovery. The LAS test was conducted using an 8 mm plate and 2 mm gap at 25 °C and 10 Hz, with strain increasing from 0.1% to 30%. These tests were used to characterize the viscoelastic response, rutting resistance, elastic recovery, and fatigue performance of the binders under different loading conditions [19].

### 3.5. Bending Beam Rheometer (BBR)

Low-temperature rheological properties were measured using a BBR (BBR B216, Matest, Arcore, Italy) according to AASHTO T313-20 [20]. Beam specimens (127 mm × 12.7 mm × 6.35 mm) were tested at −6, −12, and −18 °C under a 980 mN load for 60 s. The creep stiffness (S) and m-value were recorded. Lower S and higher m-values indicate better low-temperature flexibility and stress relaxation ability of the asphalt binder.

## 4. Test Results and Discussion

### 4.1. Softening Point Difference

**Figure 3:**
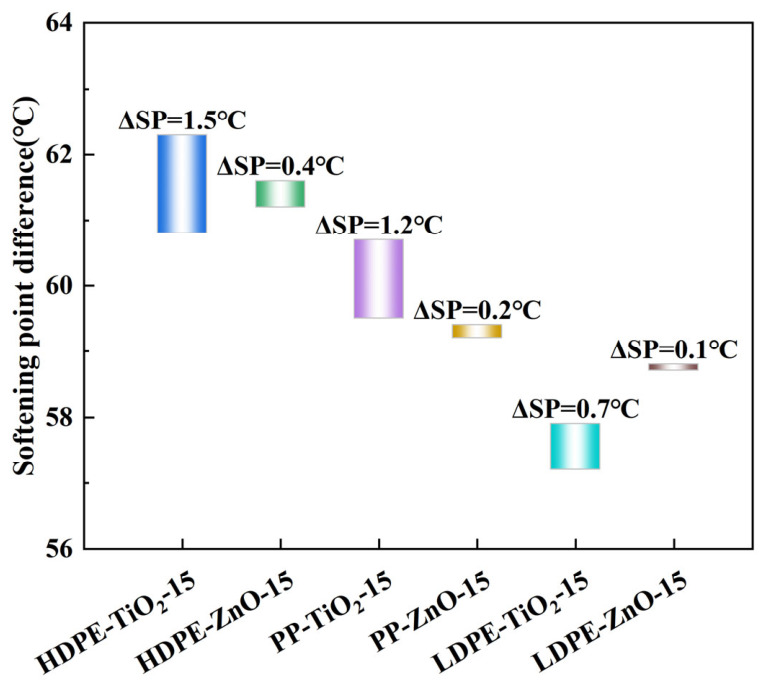
Softening point difference diagram of modified asphalt.

To evaluate the thermal storage stability and compatibility of different nanomaterial composite rubber–plastic elastomer modified asphalts, a segregation test was carried out. The softening points of the upper and lower sections after storage were measured. The difference between them, ΔSP, was used to describe the degree of phase separation under high-temperature static conditions. In general, a smaller ΔSP means that the modifier is more uniformly dispersed in asphalt. It also indicates better thermal storage stability and compatibility. In contrast, a larger ΔSP suggests that the polymer-rich phase has migrated and separated during storage [21]. As shown in Figure 3, the bar lengths of all six modified asphalts are relatively short. This means that the softening point differences are small. All ΔSP values are lower than the commonly accepted limit of 2.5 °C. This result indicates that the prepared nano rubber–plastic elastomer modified asphalts have good thermal storage stability, and no serious segregation occurred. In addition, the upper ends of the bars are generally higher than the lower ends. This means that the softening point of the upper section is slightly higher than that of the lower section after storage. It suggests that the polymer-rich phase still has a slight upward migration tendency at high temperature, but the extent is limited.

From Figure 3, clear differences can still be found among the modified systems. For the same plastic system, the bars of the ZnO-modified samples are shorter than those of the corresponding TiO_2_-modified samples. This suggests that, under the present test conditions, the ZnO-modified samples tended to show lower softening point differences than the corresponding TiO_2_-modified samples. For example, the upper and lower softening points of HDPE-TiO_2_-15 are about 62.0 °C and 60.8 °C, respectively, and its ΔSP is about 1.2 °C. For HDPE-ZnO-15, the upper and lower softening points are about 61.6 °C and 61.2 °C, and the ΔSP is about 0.4 °C. The ΔSP of PP-TiO_2_-15 is about 1.2 °C, while the ΔSP of PP-ZnO-15 is about 0.2 °C. The ΔSP of LDPE-TiO_2_-15 is about 0.7 °C. LDPE-ZnO-15 shows the smallest value, at only about 0.1 °C. When different plastic types are compared under the same nanomaterial condition, overall storage stability follows the order of LDPE system > PP system > HDPE system. In the ZnO systems, the softening point difference follows the order of LDPE-ZnO-15 < PP-ZnO-15 < HDPE-ZnO-15. In the TiO_2_ systems, LDPE-TiO_2_-15 still shows a relatively small ΔSP, while HDPE-TiO_2_-15 and PP-TiO_2_-15 show larger differences. These results suggest that both waste plastic type and nanoparticle type may have important influences on the compatibility and thermal storage behavior of rubber–plastic composite modified asphalt.

These differences are mainly related to the molecular structure of the plastics, the interfacial interactions, and the dispersion effect of the nanoparticles. LDPE has a highly branched molecular structure and better chain flexibility [22]. It can form a more uniform and stable dispersed structure in asphalt. As a result, the migration of the polymer-rich phase during storage is reduced, and the ΔSP becomes smaller. The PP system shows intermediate dispersion and stability. HDPE has a more regular molecular chain and a stronger crystallization tendency. It is more likely to form relatively independent polymer-rich domains [23]. ZnO has strong surface activity and interfacial adsorption, which can enhance the relationship between rubber phase, plastic phase and continuous asphalt phase to a certain extent, thus weakening the thermal migration and segregation of modifiers [24]. In contrast, TiO_2_ has a weaker effect on the stability of the interface, so its corresponding sample usually has a larger ΔSP value. Among the tested formulations, LDPE-ZnO-15 showed the smallest ΔSP and thus exhibited the most favorable compatibility and storage-stability trend under the present test conditions.

### 4.2. Fluorescence Microscopy Analysis

Fluorescence microscopy was used to characterize the micro-phase structure and dispersion behavior of the nanomaterial composite rubber–plastic elastomer modified asphalts [25]. In the fluorescence images, the rubber–plastic polymer phase appeared as bright green regions, while the asphalt matrix appeared as a dark background. Therefore, the compatibility and dispersion state of the modifiers could be evaluated from the size, shape, and distribution of the bright phase [26].

**Figure 4:**
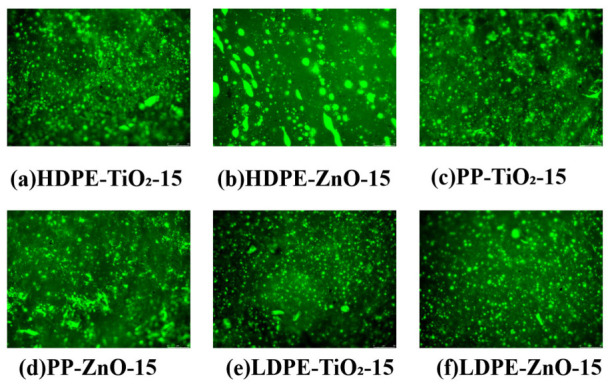
Fluorescence micrographs of modified asphalt binders.

As shown in Figure 4, the polymer phase could be observed in the asphalt matrix of all samples, but the morphology varied significantly. The HDPE-TiO_2_-15 and HDPE-ZnO-15 samples exhibited relatively large fluorescent particles together with local strip-like bright regions. This phenomenon was more evident in HDPE-ZnO-15, indicating local enrichment and coarsening of the polymer phase. Compared with the HDPE-based systems, the PP-TiO_2_-15 and PP-ZnO-15 samples showed finer bright regions, although some local aggregation still remained. By comparison, the LDPE-TiO_2_-15 and LDPE-ZnO-15 samples displayed smaller particles and a more uniform distribution. Among them, LDPE-ZnO-15 showed the most homogeneous dotted dispersion pattern, suggesting a more stable phase structure and better compatibility.

HDPE has a more regular molecular chain and a stronger crystallization tendency, so it is more likely to form independent polymer-rich regions in asphalt. LDPE has a more branched structure and better chain flexibility, which favors a finer and more uniform dispersion. PP showed intermediate behavior. The effects of ZnO and TiO_2_ were also different. ZnO improved the dispersion in the LDPE system, but it also promoted local enrichment in the HDPE system. TiO_2_ showed a relatively milder regulation effect and usually led to a medium-sized dispersed structure. These results indicate that the micro-phase structure of the modified asphalt is jointly controlled by plastic type and nanoparticle type.

To improve the objectivity of the fluorescence microscopy analysis, the images were processed by green-channel extraction, threshold segmentation, and connected-component identification to obtain the equivalent domain-size distribution, area fraction of the dispersed phase, local area-fraction coefficient of variation (CV), and nearest-neighbor index for spatial uniformity, the specific calculation results are shown in Table 6. Considering that the softening point difference (ΔSP) reflects the thermal storage stability and macroscopic compatibility of the modified binders, these image-based descriptors were further coupled with the ΔSP results to construct a corrected compatibility index, (u). The corrected results showed the following order: LDPE-ZnO-15 > LDPE-TiO_2_-15 > PP-ZnO-15 > HDPE-ZnO-15 > PP-TiO_2_-15 > HDPE-TiO_2_-15. Among them, LDPE-ZnO-15 exhibited the highest corrected (u) value, indicating smaller dispersed domains, more uniform spatial distribution, and less local aggregation, and thus the most favorable phase stability and interfacial compatibility. By contrast, HDPE-TiO_2_-15 showed the lowest (u) value, suggesting larger domains, poorer dispersion uniformity, and a greater tendency toward phase coarsening and local enrichment. Overall, the quantitative image-analysis results are in good agreement with the softening point difference results, further confirming that the LDPE-based systems, especially after ZnO incorporation, are more favorable in terms of forming a stable and homogeneous dispersed structure, whereas the HDPE-based systems remain more difficult to compatibilize effectively.

### 4.3. FTIR Analysis

**Figure 5:**
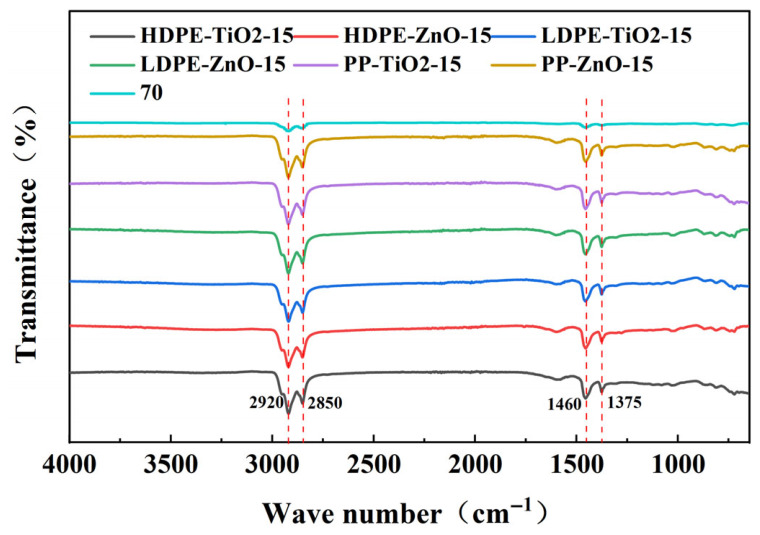
Infrared spectra of modified asphalt.

The molecular structures and modification mechanisms of the nanomaterial composite rubber–plastic elastomer modified asphalts were analyzed by FTIR. The spectra of all modified asphalts were generally similar to that of the base asphalt. As shown in Figure 5, the main absorption peaks appeared near 2920 cm^−1^, 2850 cm^−1^, 1460 cm^−1^, and 1375 cm^−1^. These bands are commonly assigned in the literature to the stretching and bending vibrations of aliphatic CH_2_ and CH_3_ groups, and similar peak positions have been widely reported for asphalt and polymer-modified asphalt systems [27]. This indicates that the modified binders still mainly retained the hydrocarbon-chain characteristics of the base asphalt and polymer modifiers [28].

The main differences among the modified systems were reflected in peak intensity and slight local peak-shape changes. No obvious new characteristic peaks or significant peak shifts were observed. This result is consistent with previous studies on non-reactive modified asphalt systems, in which the absence of new absorption bands was interpreted as evidence that the modification mainly involved physical blending, adsorption/swelling, and interfacial interaction rather than the formation of new chemical bonds [29]. Similar conclusions were also reported in studies of rubber–plastic-modified asphalt, where FTIR mainly showed the coexistence and superposition of characteristic bands from asphalt and polymer components. By contrast, some reactive modification systems reported in the literature exhibited distinct new characteristic peaks, indicating a chemical reaction between modifier and asphalt. Therefore, the present FTIR results further support that the enhancement mechanism in this study was primarily governed by physical synergistic effects and interfacial regulation.

No obvious new characteristic peaks or significant peak shifts were observed, indicating that the modification was not dominated by the formation of abundant new covalent bonds. However, this does not exclude the existence of weak interfacial interactions. The maleic anhydride compatibilizer likely acted as an interfacial bridge: its polyolefin segments were more compatible with the rubber–plastic phase, while the grafted polar groups increased the affinity of the modifier toward the more polar asphalt fractions, thereby reducing interfacial incompatibility and suppressing phase coarsening. In addition, the polar surfaces of ZnO and TiO_2_ may adsorb polar components such as resins and asphaltenes through weak Lewis acid–base interactions and possible hydrogen-bond-assisted adsorption, further enhancing interfacial adhesion and dispersion stability. Similar interfacial effects have been reported for maleic-anhydride-functionalized polyolefin modified asphalt and for asphaltene adsorption on metal oxides [30,31].

These weak interfacial interactions can help explain the rheological improvements observed in this study. Better interfacial compatibility and a finer dispersed structure can reduce phase slippage, improve stress transfer, and stabilize the modifier-rich domains, which is consistent with the improved storage stability and FM morphology. This also agrees with the superior rutting resistance and elastic recovery of the ZnO-containing systems, as well as the more favorable low-temperature response of the TiO_2_-containing systems under the present test conditions.

These results indicate that the modification mechanism was mainly dominated by physical blending, swelling adsorption, and interfacial synergistic effects. It was not dominated by the formation of new chemical bonds. The effects of plastic type mainly came from differences in molecular chain structure, while the effects of ZnO and TiO_2_ were mainly related to interfacial enhancement, filling, and dispersion regulation. Therefore, the FTIR results confirm that nanomaterial composite rubber–plastic elastomer modification did not change the basic chemical functional groups of asphalt, but improved the system through physical synergistic interactions.

### 4.4. Multiple Stress Creep Recovery Test

**Figure 6:**
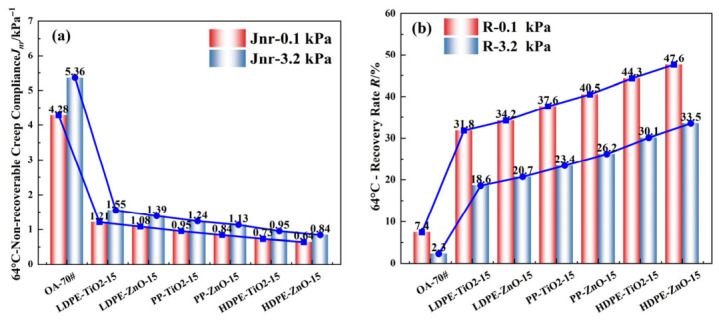
MSCR results of modified asphalt binders: (**a**) *J_nr_* and (**b**) R.

The high-temperature deformation resistances and elastic recoveries of the nanomaterial composite rubber–plastic elastomer modified asphalts were evaluated by the MSCR test at 64 °C. Two stress levels, 0.1 kPa and 3.2 kPa, were applied. Non-recoverable creep compliance (*J_nr_*) and recovery rate (*R*) were used to assess the high-temperature performance. A lower *J_nr_* indicates better resistance to permanent deformation, while a higher *R* indicates better elastic recovery [32,33]. As can be seen from Figure 6a,b, the results showed that the base asphalt had much higher Jnr values and much lower *R* values than all modified asphalts. This indicates that the nanomaterial composite rubber–plastic elastomer can significantly improve the high-temperature stability of asphalt. When the stress level increased, *J_nr_* increased and *R* decreased for all samples. This means that a higher stress level led to stronger irreversible deformation and weaker structural recovery. However, the modified asphalts showed much smaller changes than the base asphalt, which indicates better resistance to stress damage. In general, the HDPE-based systems showed the most favorable high-temperature performance trend among the tested formulations, followed by the PP-based systems, whereas the LDPE-based systems appeared relatively less favorable under the present test conditions. Within the same plastic system, the ZnO-modified samples generally appeared to show more favorable MSCR responses than the corresponding TiO_2_-modified samples under the present test conditions.

The improved high-temperature performance is mainly related to the synergistic effect of rubber, waste plastics, and nanomaterials in asphalt. The rubber phase improves elastic recovery. The plastic phase increases stiffness and structural support at high temperature. The nanoparticles improve the dispersion and interfacial interaction of the different phases. HDPE has a more regular chain structure and higher crystallinity, so it can form a more stable reinforcing skeleton in asphalt. LDPE has a more branched structure and weaker structural support, so its resistance to permanent deformation is lower. PP shows intermediate behavior. ZnO also has stronger surface activity and better interfacial regulation ability than TiO_2_. Therefore, it appeared to be more favorable for improving the high-temperature rheological response under the investigated conditions. Overall, HDPE-ZnO-15 showed the most favorable MSCR performance trend among the tested binders.

### 4.5. Linear Amplitude Scanning Test

**Figure 7:**
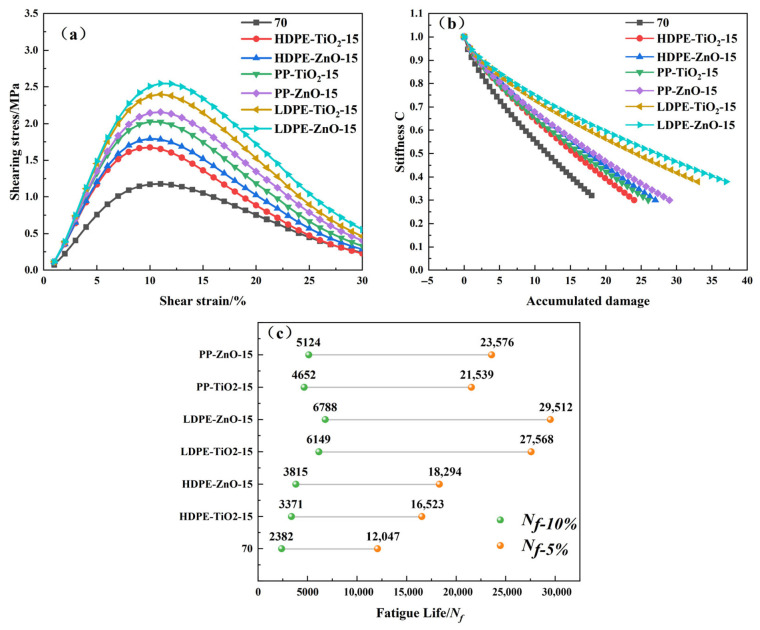
LAS test results of modified asphalt: (**a**) stress–strain curve; (**b**) damage failure curve; (**c**) fatigue life.

The fatigue behavior of the nanomaterial composite rubber–plastic elastomer modified asphalts was evaluated by the LAS test. The fatigue response was analyzed based on the viscoelastic continuum damage theory. Figure 7a shows the shear stress–strain curves. Figure 7b shows the relationship between stiffness (C) and accumulated damage (S). Figure 7c presents the predicted fatigue life (Nf) at different strain levels. These results were used to evaluate the fatigue damage evolution and cracking resistance of the modified asphalts [34,35].

As shown in Figure 7a, the shear stress of all samples first increased and then decreased with increasing strain. This indicates that the material first developed load-bearing capacity and then gradually entered the damage growth stage. All modified asphalts showed higher peak shear stress than the base asphalt. Among them, LDPE-ZnO-15 and LDPE-TiO_2_-15 showed the highest peak values. Figure 7b shows that the stiffness of all samples gradually decreased with increasing damage. However, the modified asphalts showed a slower stiffness reduction than the base asphalt. They also maintained higher stiffness at larger damage levels. Figure 7c further shows that all modified asphalts had much longer fatigue life than the base asphalt at both 10% and 5% strain levels. The fatigue life followed the order of LDPE-ZnO-15 > LDPE-TiO_2_-15 > PP-ZnO-15 > PP-TiO_2_-15 > HDPE-ZnO-15 > HDPE-TiO_2_-15.

These results indicate that the nanomaterial composite rubber–plastic elastomer can significantly improve the fatigue resistance of asphalt. The improvement is reflected by higher peak stress, slower stiffness degradation, and longer fatigue life. The LDPE-based systems showed the best fatigue performance because their flexible molecular chains help disperse local stress and delay crack growth. The PP-based systems showed intermediate performance. The HDPE-based systems improved stiffness, but their deformation coordination ability was relatively weaker. In addition, the ZnO-modified samples generally performed better than the corresponding TiO_2_-modified samples. This suggests that ZnO may provide a more favorable contribution to interfacial enhancement and damage suppression under the investigated conditions. Overall, LDPE-ZnO-15 showed the most favorable fatigue-performance trend among the tested binders.

### 4.6. Rheological Test of Low-Temperature Bending Beam

**Figure 8:**
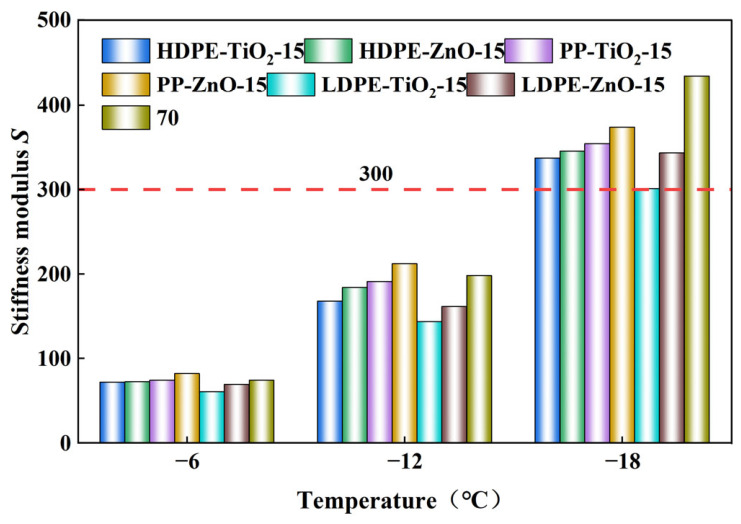
Stiffness modulus *S* value of different modified asphalts at different temperatures.

**Figure 9:**
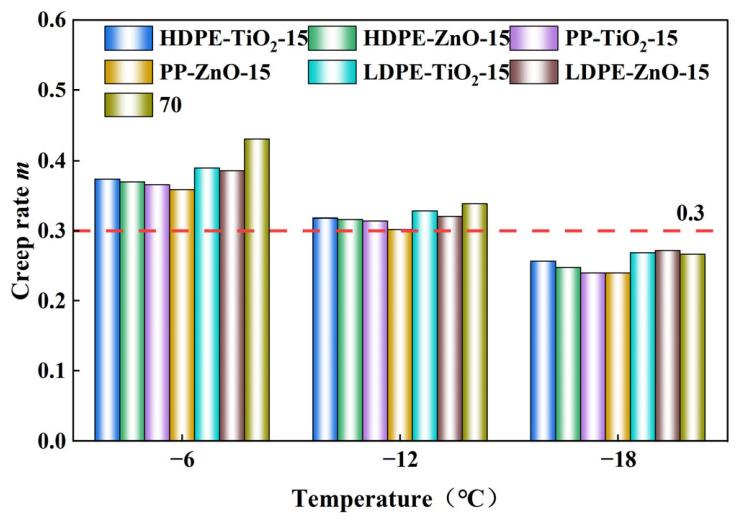
Stiffness modulus *m*-value of different modified asphalts at different temperatures.

The low-temperature rheological properties of the nanomaterial composite rubber–plastic elastomer modified asphalts were evaluated by the BBR test. The base asphalt and all modified samples were tested at −6 °C, −12 °C, and −18 °C. The creep stiffness (*S*) and *m*-value at 60 s were measured. A lower *S* value indicates better low-temperature flexibility, while a higher m-value indicates better stress relaxation and crack resistance. In general, *S* ≤ 300 MPa and *m* ≥ 0.300 are used as the criteria for acceptable low-temperature performance. As the temperature decreased from −6 °C to −18 °C, the *S* values of all samples increased, while the m-values decreased. This means that the binders became stiffer and less able to relax stress at lower temperature. In Figure 8 and Figure 9, at −6 °C and −12 °C, most samples still show acceptable low-temperature performance. At −18 °C, the crack resistance of most samples decreased significantly [36].

Clear differences were observed among the modified systems. The LDPE-based systems generally showed lower *S* values and higher m-values than the HDPE-based and PP-based systems. Among all tested samples, LDPE-TiO_2_-15 demonstrated the most favorable low-temperature cracking-resistance trend. This finding suggests that TiO_2_ may be more beneficial for improving low-temperature performance under the present test conditions, whereas ZnO appears to contribute more favorably to storage stability, rutting resistance, and fatigue-related response. For the same plastic type, the TiO_2_-modified samples usually showed lower *S* values and higher m-values than the corresponding ZnO-modified samples. This suggests that, under the present test conditions, the TiO_2_-modified samples tended to show more favorable low-temperature rheological responses than the corresponding ZnO-modified samples.

These differences are mainly related to the molecular structure of the plastics and the interfacial effect of the nanoparticles. LDPE has a more branched and flexible molecular chain, which helps form a more flexible network in asphalt and reduces thermal stress concentration. HDPE has a more regular chain structure and a stronger crystallization tendency, so it increases stiffness but weakens low-temperature deformation coordination. PP has higher chain rigidity because of its methyl side groups, so it is more likely to become brittle at low temperature. In addition, TiO_2_ was more effective in improving phase dispersion and interfacial coordination, which helped reduce local stress concentration and improve stress relaxation. Therefore, LDPE-TiO_2_-15 showed a relatively more favorable low-temperature response within the investigated systems.

As the temperature decreased from −6 °C to −18 °C, all binders showed increased stiffness and reduced m-value, indicating weakened stress-relaxation ability. However, temperature sensitivity differed among the polymer systems. The LDPE-based binders exhibited lower stiffness and higher m-values, which can be attributed to the branched and more flexible molecular structure of LDPE, allowing better deformation coordination and lower sensitivity to embrittlement [13]. In contrast, HDPE, with its more regular chains and higher crystallinity, tended to increase rigidity and hinder stress relaxation, while PP showed a greater tendency toward brittle response because of its higher chain rigidity. TiO_2_ appeared more favorable than ZnO for low-temperature performance, likely because its milder interfacial coordination effect helped reduce local stress concentration and promote stress relaxation [37]. Therefore, LDPE-TiO_2_-15 showed a relatively more favorable low-temperature response within the investigated systems.

The combined results of ΔSP, fluorescence microscopy, FTIR, and rheological tests reveal a clear structure–property relationship. HDPE-based binders tended to form coarser but more rigid modifier-rich domains because of the higher crystallinity and more regular chain structure of HDPE, which helped build a stronger high-temperature reinforcing skeleton and thus improved MSCR performance. In contrast, the LDPE-based binders showed finer and more homogeneous dispersion with lower ΔSP values, indicating better interfacial compatibility and phase stability. Owing to the branched and more flexible molecular structure of LDPE, these systems could better accommodate deformation, reduce local stress concentration, and delay crack growth, which explains their superior fatigue and low-temperature behavior. ZnO also provided stronger interfacial stabilization than TiO_2_, as reflected by the lower ΔSP values and better rutting/fatigue performance of the corresponding systems, whereas TiO_2_ showed a milder coordination effect that was more favorable for low-temperature response [38].

## 5. Conclusions

In this research, nano-reinforced rubber–plastic thermoplastic elastomer (TPE) pellets were first prepared by twin-screw extrusion using low Mooney reclaimed rubber, waste plastics, nano ZnO/nano TiO_2_, and a compatibilizer, and then incorporated into base asphalt by external addition. The storage stability, interfacial characteristics, and rheological properties of the modified asphalts were systematically investigated by softening point difference, fluorescence microscopy, FTIR, MSCR, LAS, and BBR tests. The main conclusions are as follows:

(1) A prefabricated nano-reinforced rubber–plastic TPE route was demonstrated to be feasible for asphalt modification. All six modified binders showed softening point differences lower than 2.5 °C, indicating acceptable thermal storage stability, and LDPE-ZnO-15 exhibited the smallest ΔSP among the tested formulations.
(2) The fluorescence microscopy and FTIR results consistently indicated that the enhancement mechanism was mainly attributed to phase morphology optimization and interfacial synergistic effects, rather than the formation of new chemical functional groups. Among the three plastic systems, the LDPE-based binders exhibited the finest and most homogeneous dispersed structure, followed by the PP-based systems, whereas the HDPE-based systems showed relatively coarse polymer-rich domains. No obvious new FTIR characteristic peaks were detected after modification, confirming that the modification process was dominated by physical blending, swelling/adsorption, and interfacial regulation.
(3) The nano-reinforced rubber–plastic TPE systems improved the rheological performance of asphalt to different extents. Within the investigated formulations, HDPE-ZnO-15 showed the most favorable rutting-resistance trend in the MSCR test, whereas LDPE-ZnO-15 exhibited the most favorable fatigue-related response in the LAS test.
(4) The low-temperature behavior was strongly dependent on both plastic type and nano-oxide type. Among the tested binders, LDPE-TiO_2_-15 showed the most favorable low-temperature cracking-resistance trend based on the BBR results.
(5) Overall, the results demonstrate a clear synergistic matching relationship between plastic type and nanoparticle type within the selected formulation and dosage framework used in this study. From an engineering application perspective, and within the investigated formulation framework, HDPE-ZnO-15 appears more suitable for pavements requiring enhanced high-temperature rutting resistance, LDPE-ZnO-15 appears more appropriate for conditions where fatigue durability is a key concern, and LDPE-TiO_2_-15 appears more advantageous for applications with higher demands for low-temperature crack resistance.

From an engineering viewpoint, HDPE-ZnO-15 is more suitable for hot-climate and heavy-traffic pavements, LDPE-ZnO-15 is more appropriate for applications requiring balanced durability and fatigue resistance, and LDPE-TiO_2_-15 is more advantageous for cold-region pavements with high low-temperature cracking risk. Moreover, the prefabricated TPE pellet route used in this study shows practical promise because it provides acceptable storage stability and can be implemented through a relatively conventional asphalt blending process.

This study also has some limitations. First, the material scope was relatively limited, including only one 70# base asphalt, three waste plastic types (HDPE, LDPE, and PP), two nanoparticle types (ZnO and TiO_2_), and a fixed modifier formulation. Thus, the applicability of the present findings to other asphalt sources, modifier contents, and material combinations still needs further verification. Second, this work focused on binder-scale laboratory characterization. Although the interfacial features and rheological properties were systematically evaluated, mixture-scale performance, long-term aging resistance, moisture susceptibility, and field service behavior were not considered. In addition, the conclusions are mainly based on comparative trend analysis at the binder level. More repeated testing with statistical evaluation is still needed in future work. Moreover, since a fixed TPE dosage of 15 wt.% was adopted for all formulations, the current results should be interpreted only within this dosage framework rather than as universal optimum compositions. Further studies are needed on dosage-dependent behavior, percolation-threshold-related effects, and cost-effectiveness.

## Figures and Tables

**Table 1:** Performance index of matrix asphalt.

Test Type	Test Result	Specification	Test Method
Penetration (25 °C, 0.1 mm)	66.7	60–80	T0504
Softening Point (°C)	46	≥46	T0606
Ductility (10 °C, cm)	30	≥10	T0605
Density (g/cm^3^)	1.039	-	T0603
Dynamic Viscosity (60 °C, Pa·s)	210	≥180	T0620
Flash Point (°C)	>300	≥260	T0611
Solubility (%)	99.95	≥95	T0607

**Table 2:** Low Mooney rubber performance index.

Test Type	Test Result	Specification
Heating Loss (%)	0.56	≤1.0
Density (g/cm^3^)	1.15	≤1.22
Carbon Black Content (%)	28	≥22
Rubber Hydrocarbon Content (%)	48	≥44
Mooney Viscosity	40	—

**Table 3:** Plastic performance index.

Composition	Polypropylene	High-Density Polyethylene	Low-Density Polyethylene
Density (g/cm^3^)	0.90~0.91	0.94~0.96	0.91~0.93
Melting Point (°C)	164~170	130~137	105~115
Crystallinity (%)	50~70	80~90	40~50
Stability	Good	Good	Good

**Table 4:** Performance index of inorganic nanoparticles.

Inorganic Nanoparticles	Average Particle Size	Purity (%)	Specific Surface Area (m^2^/g)	Bulk Density (g/cm^3^)	Color
ZnO	20 nm	99.9~99.99	100	0.3	White
TiO_2_	20 nm	99.9~99.99	60	0.5	White

**Table 5:** Nano rubber plastic TPE material ratio.

Number	TPE Material Ratio	Naming of Modified Asphalt
1	70% low Mooney rubber + 30% HDPE + 5% compatibilizer + 30% ZnO	HDPE-ZnO-15
2	70% low Mooney rubber + 30% HDPE + 5% compatibilizer + 30% TiO_2_	HDPE-TiO_2_-15
3	70% low Mooney rubber + 30% PP + 5% compatibilizer + 30% ZnO	PP-ZnO-15
4	70% low Mooney rubber + 30% PP + 5% compatibilizer + 30% TiO_2_	PP-TiO_2_-15
5	70% low Mooney rubber + 30% LDPE + 5% compatibilizer + 30% ZnO	LDPE-ZnO-15
6	70% low Mooney rubber + 30% LDPE + 5% compatibilizer + 30% TiO_2_	LDPE-TiO_2_-15

**Table 6:** Quantitative evaluation results of fluorescence microscopy.

Sample Number	Number of Connected Particles	Dispersed Phase Area Fraction (%)	Average Equivalent Diameter (px)	D50 (px)	Local Area Fraction CV	NNI	(u) Value
HDPE-TiO_2_-15	244	12.25	4.31	3.91	0.286	1.263	28.1
HDPE-ZnO-15	139	12.02	5.63	4.89	0.428	1.276	55.9
PP-TiO_2_-15	202	11.8	4.66	4.37	0.33	1.276	36.3
PP-ZnO-15	180	11.08	4.67	4.37	0.377	1.188	66.6
LDPE-TiO_2_-15	301	10.46	3.79	3.57	0.28	1.288	69.1
LDPE-ZnO-15	267	10.5	4.04	3.91	0.257	1.322	98.2

## Data Availability

The original contributions presented in this study are included in the article. Further inquiries can be directed to the corresponding author.

## Abbreviations

Abbreviation	Full Name
HDPE	High-Density Polyethylene
LDPE	Low-Density Polyethylene
PP	Polypropylene
TPE	Thermoplastic Elastomer
ZnO	Zinc Oxide
TiO_2_	Titanium Dioxide
FTIR	Fourier-Transform Infrared Spectroscopy
AFM	Atomic Force Microscopy
BBR	Bending Beam Rheometer
LAS	Linear Amplitude Sweep
MSCR	Multiple Stress Creep Recovery
DSR	Dynamic Shear Rheometer
LCC	Life Cycle Cost
